# Modelling individual tree height to crown base of Norway spruce (*Picea abies* (L.) Karst.) and European beech (*Fagus sylvatica* L.)

**DOI:** 10.1371/journal.pone.0186394

**Published:** 2017-10-19

**Authors:** Ram P. Sharma, Zdeněk Vacek, Stanislav Vacek, Vilém Podrázský, Václav Jansa

**Affiliations:** 1 Faculty of Forestry and Wood Sciences, Czech University of Life Sciences Prague, Prague, Suchdol, Czech Republic; 2 Krkonoše Mountains National Park Administration, Department of Nature Conservation Dobrovského, Vrchlabí, Czech Republic; Ecole Pratique des Hautes Etudes, FRANCE

## Abstract

Height to crown base (HCB) of a tree is an important variable often included as a predictor in various forest models that serve as the fundamental tools for decision-making in forestry. We developed spatially explicit and spatially inexplicit mixed-effects HCB models using measurements from a total 19,404 trees of Norway spruce (*Picea abies* (L.) Karst.) and European beech (*Fagus sylvatica* L.) on the permanent sample plots that are located across the Czech Republic. Variables describing site quality, stand density or competition, and species mixing effects were included into the HCB model with use of dominant height (HDOM), basal area of trees larger in diameters than a subject tree (BAL- spatially inexplicit measure) or Hegyi’s competition index (HCI—spatially explicit measure), and basal area proportion of a species of interest (BAPOR), respectively. The parameters describing sample plot-level random effects were included into the HCB model by applying the mixed-effects modelling approach. Among several functional forms evaluated, the logistic function was found most suited to our data. The HCB model for Norway spruce was tested against the data originated from different inventory designs, but model for European beech was tested using partitioned dataset (a part of the main dataset). The variance heteroscedasticity in the residuals was substantially reduced through inclusion of a power variance function into the HCB model. The results showed that spatially explicit model described significantly a larger part of the HCB variations [R^2^_adj_ = 0.86 (spruce), 0.85 (beech)] than its spatially inexplicit counterpart [R^2^_adj_ = 0.84 (spruce), 0.83 (beech)]. The HCB increased with increasing competitive interactions described by tree-centered competition measure: BAL or HCI, and species mixing effects described by BAPOR. A test of the mixed-effects HCB model with the random effects estimated using at least four trees per sample plot in the validation data confirmed that the model was precise enough for the prediction of HCB for a range of site quality, tree size, stand density, and stand structure. We therefore recommend measuring of HCB on four randomly selected trees of a species of interest on each sample plot for localizing the mixed-effects model and predicting HCB of the remaining trees on the plot. Growth simulations can be made from the data that lack the values for either crown ratio or HCB using the HCB models.

## Introduction

Growth capacity of a tree is determined by its foliage surface area or foliage volume, but because of measuring difficulty of these variables, crown length or relative crown length (crown ratio) are commonly used as surrogates in various growth and yield models [1]. Foliage surface area and foliage volume including crown ratio (CR) are related to the assimilation and release of energy, photosynthesis, and transpiration in a tree [2, 3]. The CR is a ratio of crown length to total height of a tree with values ranging from 0 (for a tree without crown or defoliated tree, i.e., growth constrained by lack of foliage) to 1 (for a tree crown extending over the entire tree trunk, i.e., maximum leaf area or foliage mass attainable for a tree of a given height) [4]. Analysis of crown dimensions including CR is important for quantifying and qualifying tree vigor, competition, growth stage, and stability and production efficiency, and CR is a good indicator of the tree vigor [5–7], wood quality [8, 9], and wind firmness [10]. The CR has widely been used as an important predictor in forest growth and yield models [11–17], and tree taper models [18]. The CR may be used for management of recreational forests and wildlife habitats [19, 20].

In growth simulations, where projections for a given period are based on the stand and tree variables at the beginning of that period, CR of the growth model has to be updated for each successive projection period [1]. If the crown change cannot be predicted directly [21], static models for predicting height to crown base (HCB) can be used to simulate this change [14, 15, 22]. The static crown models can also replace missing crown measurements so that growth simulations can be made from the data that lack the values of CR or HCB [1]. Crown length, HCB, bole length, CR, and bole ratio are algebraically interrelated. In order to estimate CR, HCB or crown length should be measured. However, measuring the HCB of each tree on a sampled area is relatively difficult, time-consuming, and costly, especially for dense and multi-layered stands [4, 23], and prone to errors due to conflicting definitions [24], and therefore, measurements are often limited. Alternatively, HCB models also called static HCB models developed with the extensive data can be used for a precise prediction of the missing HCB measurement [4, 25, 26] and estimation of the changes in CR [14]. Static approach is more commonly used over the dynamic ones, because of lack of the repeated measurements of the HCB and errors associated with various HCB definitions [24, 27]. In recent years, many studies have developed the crown length models [28, 29], crown ratio models [5, 23, 30–32] that can be used as decision-support tools in forest management.

A number of HCB models for a variety of tree species have already been developed by applying simple to complex modelling approaches [1, 4, 25, 27, 33, 34] and the predictors used in these models are diameter at breast height, total height, diameter-height ratio, crown competition factor, stand basal area, and site index or dominant height. However, none of them has used spatially explicit (distance dependent) competition measure to describe competitive interactions among the individual trees. A forest stand is an aggregate of the individuals competing over the restricted distance, and competition largely influences growth, mortality, and regeneration [35–37]. Spatial pattern of the individuals may change from aggregate to regular form in course of stand development and crown gradually expands to a full occupancy of site, but this pattern may change due to mortality and management interventions [38]. Thus, the competition measures computed taking into account the spatial position of all individual trees better describe competitive interactions among them [36, 37, 39–41]. Even though previous HCB modelling studies are based on the mixed species stands, none of them has considered the species mixing effects on the HCB models. The effect of species mixture on tree growth and stand dynamics is significantly high [42–47], and therefore this should be included into the forest models when data from the mixed species stands are used.

To the authors’ knowledge, except three HCB studies [4, 25, 27], all others have developed HCB models applying the ordinary least square regression which is not a suitable method for hierarchically structured data. Repeated measurements from the same tree or measurements from multiple trees on the same sample plot are significantly correlated to each other [4, 48–50]. When the ordinary least squares regression is applied to estimate the model using these data, the assumption of independent errors is largely violated and estimated parameters and variances are significantly biased [48, 50, 51]. The mixed-effects modelling approach needs to be applied to deal with this problem as this analyzes the hierarchically structured data more efficiently, and increases the model’s prediction accuracy [50, 52–54].

The objectives of this study are to (i) develop the mixed-effects HCB models using total height, diameter at breast height, dominant height, tree-centered competition measures (basal area of trees larger in diameters than a subject tree and Hegyi’s competition index), basal area proportion of a species of interest as fixed predictors, and sample plot-level variations as random effects; (ii) compare the mixed-effects HCB model developed using spatially explicit competition measure (Hegyi’s competition index) with the mixed-effects HCB model developed using spatially inexplicit competition measure (basal area of trees larger in diameters than a subject tree); and (iii) determine an optimal number of trees per sample plot for localizing the mixed-effects model and precise prediction of HCB for the remaining trees on the sample plot. A set of variables that describe the effects of size and vigor of trees, site quality, and stand density were measured on the permanent sample plots located across the Czech Republic. Measurements from a total of 19,404 trees were used for modelling. Majority of modelling data originated from mixed species stands. The proposed HCB models can be used to accurately predict the missing measurements of HCB for trees of Norway spruce (*Picea abies* (L.) Karst.) and European beech (*Fagus sylvatica* L.) in subsequent inventories of the permanent sample plots, so that growth simulations can be made from the data that lack the values for either CR or HCB.

## Materials and methods

Our field inventory and research did not involve any endangered or protected species of plants and animals. All measurements were carried out in accordance with the notification provision of protection of the nature, and therefore not detrimental to wildlife, soil and plant resources. In this study, we used two extensive datasets: training dataset (model fitting dataset) and model validation dataset, in order to develop and validate the HCB models, respectively. A validation dataset of Norway spruce (*Picea abies* (L.) Karst.) was collected from different sampling designs and locations, and therefore it had different characteristics and coverage of growing conditions than those of the training dataset. However, a validation dataset of European beech (*Fagus sylvatica* L.) was a partitioned dataset (a part of the main dataset), and therefore it was similar to the training dataset. We briefly describe both datasets in the following sub-sections, and readers may get access to these datasets in a supporting information: S1 Table in this article.

### Training dataset

**Fig 1:**
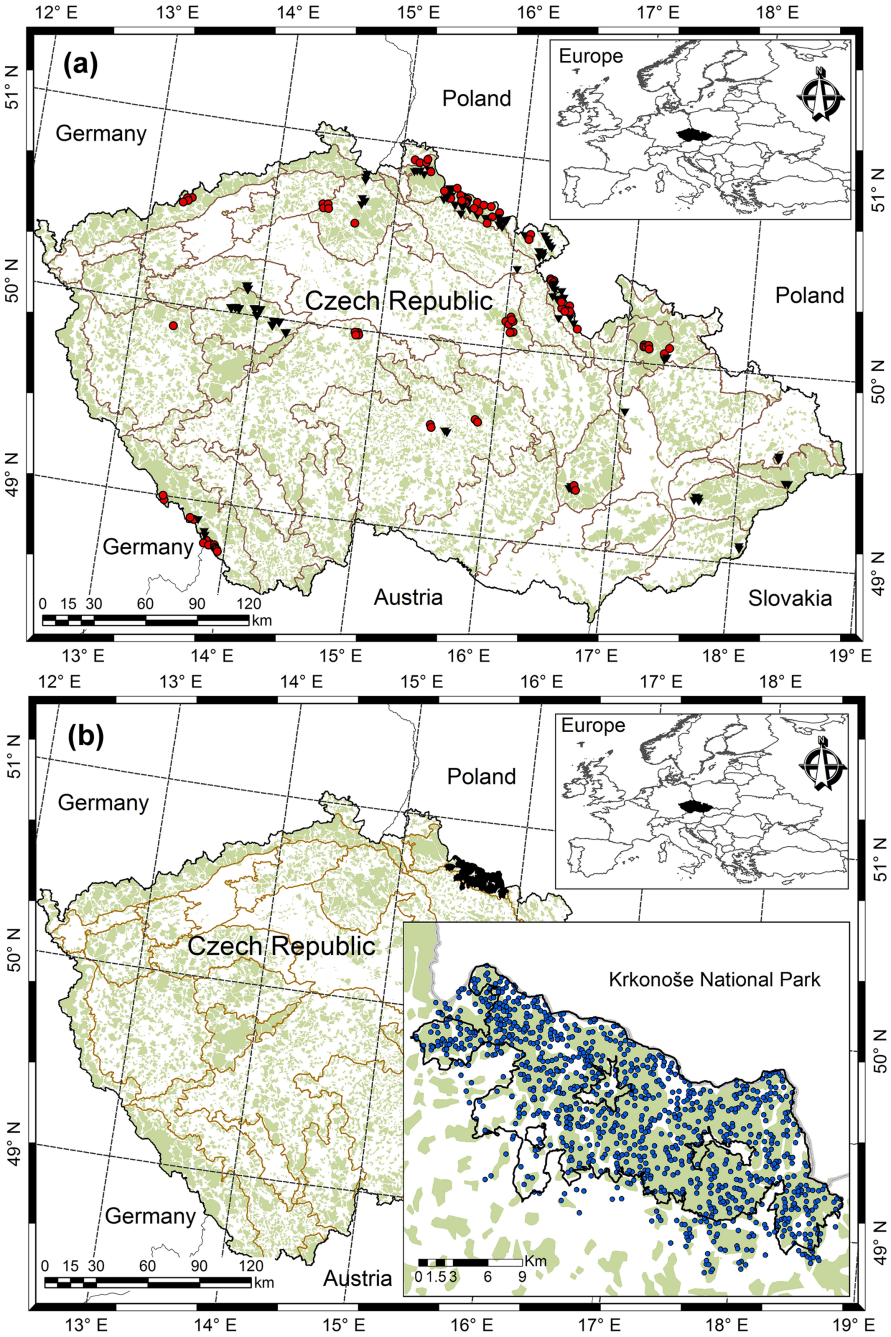
Location of sample plots. (**a**) training data [purely Norway spruce or Norway spruce-dominated sample plots (red dots), purely European beech or European beech-dominated sample plots (black triangles), light green dots showing forest cover, and grey lines separating Natural Forest Area], (**b**) model validation data collected on the sample plots in the Krkonoše national park.

A training dataset was collected from 174 permanent research plots, hereafter termed as sample plots (124 plots for Norway spruce, 50 plots for European beech), which are located in various parts of the Czech Republic (Fig 1a). The squared-shaped sample plots with size varying from 2500 m^2^ to 4900 m^2^ were established in the stands by considering some important criteria, such as canopy structure, mortality and regeneration, and stocking of dead woods. Sample plots represent a wide variability of site quality, stand density, species composition, stand development stage, and management regime. A sample plot network also covers a wide range of altitude (240 m—1370 m), mean annual temperature (4–9.5°C), mean annual precipitation (500–1450 mm), and growing season length (45–180 days). Growing season length was defined as the number of days in a year when mean daily temperature was above 10°C, and all mean values of climate variables were based on the climate records between 1963 and 2012. Most of the stands, especially European beech originated from the natural regeneration and about 20% Norway spruce from the plantation. About 77% stands aged 20 to 150 years were left for natural development where management was based on the minimum harvesting approach, and this included salvage cutting and sanitary interventions, e.g. extraction of the trees affected by bark beetles and diseases. Management of the rest of the studied forests mainly focused on the shelter wood selection that involved 5% gap formation within a stand. We excluded those sample plots, which were severely affected by disturbances. More detailed descriptions of these sample plots can be found in the literatures [46, 55–58]. The training dataset comprised 19% and 18% monospecific sample plots for Norway spruce and European beech, respectively. Definition of the monospecific stands considered the inclusion of all individuals other than a species of interest if they had DBH < 4 cm.

Following the inventory protocols developed by the Forest Management Institute [59], all measurements were made between April 2007 and March 2016. However, no repeated measurements were involved, meaning that there was no temporal variation in the data. Over-bark diameter at breast height (DBH, 1.3 m above ground) was measured with a precision of 1 mm and total height with a precision of 0.1 m. Regardless of the species of interest (Norway spruce and European beech), position of all living trees with DBH ≥ 4 cm, and regeneration with DBH < 4 cm and height ≥ 2 m was also recorded. Height to live crown base (HCB) for all individuals of Norway spruce including regeneration was measured at the lowest point on the trunk where continuous whorl of at least two living branches was formed, and this excluded epicormic and adventitious sprouts [46, 57]. However, this whorl was not considered as a crown base when there were at least three dead whorls above it. For European beech and other broadleaves, HCB was considered up to the point where the lowest continuous live crown whorl was formed. However, a whorl was not considered as a live crown base when there was a continuous whorl of newly sprouts or epicormic and adventitious sprouts growing on the trunk below the continuous live crown whorl formed by branches.

### Validation dataset

A validation dataset for Norway spruce was collected from forest stands in the Krkonoše National Park located in northern part of the Czech Republic (Fig 1b). The park with an area of 363 km^2^ was first declared in 1963 and later extended to an area of 550 km^2^ in 1968. The park was included as a bilateral Biosphere Reserve in the World Network of Biosphere Reserves in 1992. Precipitation, temperature, and growing season length substantially vary with altitude and aspect within a park, where mean annual precipitation varies from 860 to 1260 mm, mean annual temperature from 2.6°C to 6.1°C, and growing season length from 150 to 35 days [56].

Norway spruce is a dominant tree species with 87% share of the total forest cover of the park. A total of 830 circular permanent research plots, hereafter termed as sample plots, with an area of 500 m^2^, covering the entire forests of the park, were established. All measurements were made with a permission from the park authority. Over-bark DBH was measured for all individuals with DBH ≥ 7 cm with a precision of 1 mm. Total height and HCB were measured with a precision of 10 cm for at least five dominant tree species and one trees for each of the other tree species on each sample plot. The first measurement was made between May 2009 and December 2010, and second measurement between June 2012 and July 2014. However, we only used the first measurements. About 69% data in the validation set for Norway spruce originated from the monospecific sample plots. A detailed description of these spruce sample plots is found in Sharma et al. [60].

**Fig 2:**
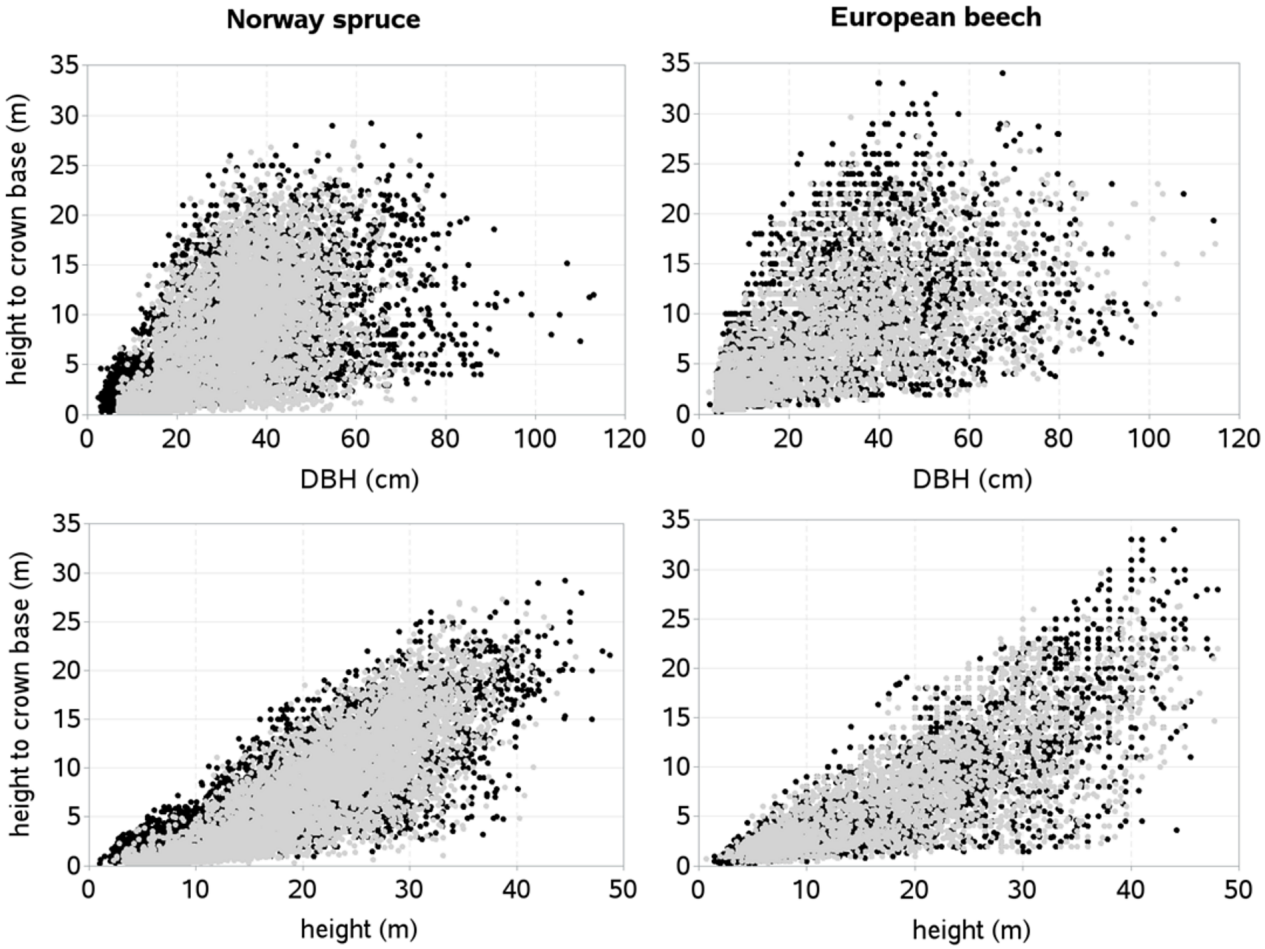
Height to crown base against diameter at breast height and total height, respectively. [Training dataset: black dots; model validation dataset: gray dots].

Because of unavailability of any external independent dataset, we divided the main dataset to validate the HCB model for European beech, and characteristics of the validation data for this species are similar to those of the training dataset. A validation dataset for this species was allocated from 48 sample plots, which were randomly selected from a total of 98 sample plots, and therefore, like training dataset, this also represents a wide variability of site quality, stand density, species mixture, stand development stage, and management regime. About 23% data in a validation set for European beech originated from monospecific sample plots. The patterns of training and validation datasets are shown in Fig 2. The general distribution patterns of HCB and predictors were also examined. Because of large datasets collected from the stands representative to all possible stand development stages, distribution patterns of HCB and other predictors seemed approximately symmetric, balanced, and regular.

### Data analysis

Data analysis involved the computation of stand measures that describe site quality, stand density, and species mixing effects. Analysis included the computation of spatially explicit competition index that describes competitive interactions among the individual trees within a specified distance around the subject trees. Analysis also included fitting and evaluation of the candidate models, and selection of the best model for further expansion and evaluation.

#### Stand variables

The influences of site quality and stand density on tree characteristics including HCB could be substantial [4, 5, 25, 46, 61, 62]. In order to evaluate the contributions to the HCB model, we computed various stand- and tree-level variables. Because of lack of site index data, we included dominant height as a proxy of site index in the HCB model to describe site quality effect. Since tree growth and stand development are strongly correlated to dominant height, this has frequently been used as a surrogate of site index in various forest models including HCB models [4, 23, 46, 57, 58, 63–65]. Following the methods suggested by Sharma et al. [46, 66], we identified dominant trees from the measured height sample trees per sample plot and calculated the dominant height (HDOM). We calculated tree-centered spatially inexplicit competition measure, i.e., basal area of trees larger in diameters than a subject tree (BAL). We also calculated various stand-level variables such as the number of stems per hectare, sum of DBH of all individuals per sample plot (DBHSUM), DBHSUM per hectare, stand basal area (BA), arithmetic mean DBH (AMD) and quadratic mean DBH (QMD), relative spacing index (RSI) -defined as a ratio of the average inter-tree distance to the dominant height [67]. The potential contributions of relative size of the trees to the HCB models such as DBH-to-QMD ratio (dq) and height-to-HDOM ratio were also evaluated. Since the effect of species mixture or inter-or intra-specific interactions on tree growth and stand dynamics is highly significant [42, 43, 46, 47, 57], this should not be neglected when data originated from mixed species stands were used to develop HCB models. We therefore calculated species proportion for each of the above-mentioned stand variables and examined their potential contributions to the HCB models.

#### Spatially explicit competition index

The coordinates of all trees regardless of species of interest on each sample plot were recorded, and therefore we were able to compute spatially explicit competition indices that describe competitive interactions among the individual trees within a certain distance. Even though height and crown dimensions might better describe competition between the trees [68, 69], missing measurements of crown width of the number of trees including species of interest did not allow us to compute the distance-weighted crown measure-ratios such as ratios of crown cross-sectional areas or crown volumes of a subject tree to its competitors. Instead, we chose the Hegyi’s index [70] as a distance-weighted DBH-ratio index (Eq 1) that quantifies the competitive stress of the neighbors located within a certain distance around a subject tree. This index is based on the assumption that larger and closer neighbors contribute higher competitive stress to a subject tree. Compared to other indices reported in the literatures [36, 39–41, 58, 68], Hegyi’s index is simpler to compute and easier to interpret.

$$HCI=\sum_{c=1}^{n}\lambda_{sc}\left(\frac{DBH_{c}}{DBH_{s}}\right)\frac{1}{DIST_{sc}}$$ (1)

where *CI* is competition index; *DBH* is diameter at breast height of a tree; *DIST* is distance between subject tree and competitor; *n* is the number of competitors of a subject tree; *λ* is edge correction factor; *s* is an index for a subject tree; and *c* is an index for a competitor.

We identified all potential competitors lying within a certain maximum distance around each subject tree by applying horizontal and vertical search angles [68, 69]. However, among several alternatives of search radii [46, 58, 68] evaluated, following vertical search angle-based alternative provided the best performance:

$$DIST_{sc}\leq \frac{H_{c}}{tan\theta }$$ (2)

where *H*_*c*_ is total height of a competitor *c*, and other acronyms and indices are the same as in Eq 1, *θ* is an angle of the inclination to the horizontal line starting from the base of a subject tree *s* to the top of a competitor *c*.

We allowed *θ* to vary from 25° to 70° by 1° increment in Eq 2 while computing HCI (Eq 1), which resulted in 45 HCI alternatives. We compared the contributions of each of these HCIs to the HCB model using model evaluation measure such as the smallest sum of square error. An index with a search angle of 50° (*θ* = 50°) for both species provided the highest contribution to description of the competitive interactions among the individual trees.

For spatially explicit competition indices, competitors located beyond the sample plot boundary are of a concern. The number of recorded competitors is systematically lower for trees closer to the sample plot boundary, leading to biased estimates of the competitive interactions [41]. Therefore, to reduce the potential errors caused by off-plot competitors, we computed the edge correction factor (*λ*) for each sample plot using the methods suggested by Martin et al. [71] and Goreaud and Pélissier [72]. The correction factor computed with these methods could be more reliable if stand conditions inside-sample plot and outside-sample plot were identical. Our stem maps did not show the intersected sample plots, and therefore this assumption could be more likely hold. The HCI and other tree- and stand-level variables for both training and validation datasets are summarized in Table 1.

**Table 1:** Data summary.

Variables	Data statistics [mean ± standard deviation (range)]			
	Norway spruce		European beech	
	Model fitting	Model validation	Model fitting	Model validation
Number of sample plots	124	830	50	48
Total number of HCB sample trees	6331	5516	3963	3594
Number of HCB trees per sample plot	170 ± 140 (4–450)	16 ± 6 (5–38)	118 ± 79 (4–301)	115 ± 75 (4–287)
Number of stems (N ha^-1^)	1013 ± 678 (92–2568)	308 ± 128 (30–750)	1030 ± 836 (32–2568)	736 ± 622 (100–2440)
Stand basal area (BA, m^2^ ha^-1^)	51.3 ± 21.6 (10–71.2)	26.9 ± 16.8 (0.1–74.8)	31.3 ± 17.8 (0.5–75.6)	47.5 ± 17.8 (0.5–84.2)
BA proportion of a tree species (BAPOR)	0.71 ± 0.3 (0.00052–1)	0.93 ± 0.14 (0.0041–1)	0.66 ± 0.22 (0.0002–1)	0.73 ± 0.24 (0.01–1)
BA of trees lager than a subject tree (BAL, m^2^ ha^-1^)	35.3 ± 19.4 (0–67.6)	23.8 ± 17.7 (0–73.1)	28.4 ± 18.5 (0–69.6)	33.2 ± 17.3 (0–71.9)
Quadratic mean DBH per sample plot (QMD, cm)	28.8 ± 9.7 (11.9–60.5)	33.4 ± 10.5 (7.4–60.3)	31.5 ± 12.7 (15.6–87.4)	33.1 ± 11.3 (8.1–82.8)
DBH-to-QMD ratio (dq)	0.89 ± 0.55 (0.11–6.04)	1.01 ± 0.52 (0.11–7.1)	0.78 ± 0.62 (0.09–5.11)	0.71 ± 0.69 (0.07–4.72)
Arithmetic mean DBH per sample plot (cm)	24.8 ± 10.6 (9.4–53.9)	28.6 ± 9.8 (6.8–56.9)	27.1 ± 14.0 (9.5–84.4)	28.4 ± 11.7 (10.6–66.3)
DBH sum per sample plot (cm)	4869 ± 1870 (813–9334)	478 ± 244 (43–1193)	4775 ± 2116 (675–9333.6)	4023 ± 1365 (1656–6511)
Dominant height per sample plot (HDOM, m)	26.9 ± 8.1 (8–42)	20.4 ± 7.8 (3.5–37.6)	31.4 ± 6.0 (19.7–42.8)	30.2 ± 7.1 (6.2–42.5)
Relative spacing index (RSI)	0.16 ± 0.07 (0.05–0.45)	0.12 ± 0.05 (0.03–0.63)	0.13 ± 0.06 (0.06–0.41)	0.15 ± 0.05 (0.06–0.93)
Total height (H, m)	16.7 ± 9.8 (2–48.7)	19.5 ± 8.4 (2.3–45.5)	18.9 ± 10.8 (2.3–48)	18.7 ± 9.9 (2.0–47.8)
H-to-HDOM ratio	0.63 ± 0.31 (0.03–1.59)	0.59 ± 0.34 (0.02–1.72)	0.61 ± 0.30 (0.06–1.41)	0.57 ± 0.33 (0.04–1.54)
Height to crown base (HCB, m)	6.6 ± 5.7 (0.2–29.2)	7.9 ± 5.7 (0.2–27.4)	8.1 ± 6.6 (0.2–34.5)	7.5 ± 5.5 (0.3–29.6)
Diameter at breast height (DBH, cm)	26.5 ± 18.4 (2.4–113)	31.9 ± 13.1 (7–76.8)	26.8 ± 20.5 (2.3–114.3)	28.01 ± 20.4 (2.3–114.6)
Hegyi’s competition index (HCI)	6.3 ± 7.8 (0–38.4)	-	6.4 ± 5.6 (0–27.2)	7.5 ± 6.6 (0–31.7)

#### Model construction

In the first stage, considering the theoretical principle that HCB never exceeds the total tree height (H), we chose various mathematical functions with use of DBH and H as main predictors, and fitted to data and evaluated their fitting performance. In the second stage, the best performing function was expanded through the integration of additional variables describing the effects of site quality, competition, species mixture, and parameters describing the sample plot-level random effects.

#### Base model

We fitted various functions presented in the literatures [1, 4, 25, 33] and evaluated using the statistical measures (to be described later). Besides H, DBH also is a key predictor in the forest growth and yield models and easily measured for practical application, and therefore these two variables were included as main predictors in the HCB models [4]. The ordinary least squares regression was used to fit the base models. Following form of the logistic model was found most suited to our data:

$$HCB_{ij}=\;\frac{H_{ij}}{\left[1+exp\left(b_{1}+b_{2}DBH_{ij}\right)\right]}+\varepsilon_{ij}$$ (3)

where *HCB*_*ij*_, *H*_*ij*_, and *DBH*_*ij*_ are height to crown base (m), total height (m) and diameter at breast height (cm) of the *j*^*th*^ tree on the *i*^*th*^ sample plot, respectively, *b*_*1*_ and *b*_*2*_ are parameters, and *ε*_*ij*_ is an error term. This functional form was also found most suited to data of different tree species in other studies [1, 4, 25, 33]. Since HCB predictions with this model could be constrained between 0 and H, it is considered the most suitable for HCB modelling.

#### Inclusion of additional predictors

The HCB is significantly affected by tree size and vigor, site quality, and stand density or competition [1, 4, 11, 18, 25, 33]. We evaluated several predictors (Table 1) that describe the effects of site quality, competition, and species mixture for their potential contributions to description of the HCB variations using Eq 3. Our evaluation was based on whether the predictors were suited to the model fitting procedure, beginning with graphical exploration of data and examination of the correlation statistics [4, 46, 73]. The interactions between variables and their transformations (logarithmic, inverse, square) were also evaluated. Highly contributing predictors identified are HDOM, BAL and BAPOR, because they exhibited significant correlations with HCB in the step-wise variable selection procedure [74, 75]. Incorporating additional predictors into the models might improve the fit statistics, but we did not do this, realizing the fact that many predictors in the model could cause over-parameterization, resulting in biased parameter estimates and variances [57, 74]. Following is the most suitable expanded model to our data:

$$HCB_{ij}=\frac{H_{ij}}{\left[1+exp\left(\begin{array}{c} b_{1}+b_{2}DBH_{ij}+b_{3}H_{ij}+b_{4}HDOM_{i}+b_{5}BAL_{ij}\\ +b_{6}BAPOR_{ik} \end{array}\right)\right]}\;+\varepsilon_{ij}$$ (4)

where *HCB*_*ij*_, *H*_*ij*_, *DBH*_*ij*,_ and *BAL*_ij_ are height to crown base (m), total height (m), diameter at breast height (cm), and basal area of trees larger in diameters than the *j*^*th*^ subject tree on the *i*^*th*^ sample plot (m^2^ ha^-1^), respectively; *HDOM*_i_ and *BAPOR*_ik_ are sample plot dominant height (m) and basal area proportion of the *k*^*th*^ species (*k* = Norway spruce or European beech) on the *i*^*th*^ *s*ample plot, respectively, *b*_*1*,*…*,_ *b*_*6*_ are parameters, and *ε*_*ij*_ is an error term. This model is henceforth termed as a spatially inexplicit HCB model, and the model with *BAL*_*ij*_ replaced by *HCI*_*ij*_ is termed as a spatially explicit HCB model.

#### Inclusion of random effects

The mixed-effects HCB model was formulated using the global HCB model (Eq 4) through the integration of the sample plot-level random effects, because this model assumes the invariability of HCB across the sample plots, which does not hold. We therefore included the parameters that describe sample plot-level random effects to determine the extent to which the subject-specific HCB model or localized model improves the prediction accuracy compared to that of the global model. The objective of inclusion of the random effect parameters into the global model is to make the developed mixed-effects model sample plot-specific and secure a high prediction accuracy [4, 48–54, 57]. All possible combinations of the random effects and fixed effect parameters in Eq 4 were fitted to the data. However, convergence was only possible with the random effect parameters combined with one or two fixed parameters of the model. The following mixed-effects model formulation, which showed the smallest Akaike’s information criterion (AIC), was selected for further evaluation:

$$\begin{array}{l} HCB_{ij}=\frac{H_{ij}}{\left[1+exp\left\{\begin{array}{c} \left(b_{1}+u_{i1}\right)+(b_{2}+u_{i2})DBH_{ij}+b_{3}H_{ij}+b_{4}HDOM_{i}\\ +b_{5}BAL_{ij}+b_{6}BAPOR_{ik} \end{array}\right\}\right]}+\varepsilon_{ij}\\ \text{with}\;\varepsilon_{i}∼N\left(0,R\right),u_{i}∼N\left(0,D\right) \end{array}$$ (5)

where all acronyms, symbols, and indices are the same as in Eq 4, ***u***_***i***_ is a vector of the random effects (*u*_*i*1_ *u*_*i*2_) of the *i*^*th*^ sample plot, and was assumed to be normally distributed with zero expectation and within-sample plot variance-covariance matrix of ***D*** defined by

$$D=\left[\begin{array}{cc} \sigma_{ui1}^{2} & \sigma_{ui1ui2}\\ \sigma_{ui1ui2} & \sigma_{ui2}^{2} \end{array}\right]$$ (6)

where $\sigma_{ui}^{2}{}_{1}$, $\sigma_{ui}^{2}{}_{2}$ and $\sigma_{ui_{1}ui_{2}}$ are variance-covariance components of *i*^*th*^ sample plot, and a matrix ***R***_***i***_ with *n*_*i*_ *× n*_*i*_ dimensions is within-sample plot variance-covariance matrix of the error term **ℰ**_***i***_, and it is defined by

$$R_{i}=\sigma^{2}G_{i}^{0.5}{Г}_{i}G_{i}^{0.5}$$ (7)

where *σ*^*2*^ is a residual variance common to all sample plots, ***G***_***i***_ with *n*_*i*_ *× n*_*i*_ dimensions is a diagonal matrix, which accounts for within-sample plot variance heteroscedasticity. The matrix ***Г***_***i***_ with *n*_*i*_ *× n*_*i*_ dimensions accounts for within-sample plot autocorrelation structure of the residual errors (*n*_*i*_ is the number of observations on the *i*^*th*^ sample plot), which was reduced to the identity matrix, ***I***_***i***_, because autocorrelations did not present in our data.

Our preliminary analyses showed that there was a variance heteroskedasticity in the data. Therefore, it was necessary to reduce this problem through the application of an appropriate variance function. We evaluated three variance functions (exponential, power, and constant plus power functions) with each of the five variables (DBH, H, HDOM, BAL, BAPOR) and relative values of HCB (HCB/H) as independent variable to stabilize the variance of the within-sample plot heteroscedasticity. We found that the power variance function with *H* as an independent variable (Eq 8) accounted for the variance heteroscedasticity most effectively.

$$var\left(\varepsilon_{i}\right)=\sigma^{2}H_{ij}^{2\varphi }$$ (8)

where, *φ* is a parameter to be estimated and *σ*^*2*^ is the same as in Eq 7. The diagonal elements of a matrix ***G***_***i***_ (Eq 7) are provided by this variance function for the application of the mixed-effects HCB model.

#### Model estimation and evaluation

All candidate base models were estimated using PROC MODEL [76] with nonlinear least square nonlinear regression method. The mixed-effects models were estimated with the restricted maximum likelihood in SAS macro NLINMIX [76] using expansion-around-zero method [77]. All model alternatives including base models were evaluated by according to their root mean squared errors (RMSE), adjusted coefficient of determination ($R_{adj}^{2}$), and Akaike’s information criterion (AIC). Formulae of these statistical measures are available in the standard textbooks of statistics such as Montgomery et al. [74]. Unless otherwise specified, we used 1% level of significance in all analyses. Even though there were identical statistical measures with two or more fitted models, the curve profiles generated with them might differ from the measured ones, resulting in a relatively bigger or smaller deviation in the residuals [57]. We therefore simultaneously examined both numerical statistical measures and graphs of the residuals plotted against each of the potential predictors and simulated HCB curves overlaid on the measured data.

We carried out the model validation, which is one of the most important tasks in modelling, as this provides the credibility and confidence about the developed model. We used the external independent data for Norway spruce and a part of the main dataset (partitioned data) for European beech, because external independent dataset for this species was not available. The model validation with an external independent dataset provides important information in addition to the respective fit statistics obtained from the training dataset [66, 78–80]. More details about the model validation with the subject-specific predictions are given in the following sub-sections.

#### Prediction with mixed effect HCB model

One of the following situations can be considered while applying the mixed effect model to predict HCB [49, 57, 65]:

1. Mean response: This is also known as a typical response or fixed-effect response or population average or mean response. This involves the prediction of HCB using only input information of the predictors used in the model (Eq 5), but no estimation of the sample plot-specific random effects (*u*_*i*1_ *u*_*i*2_) is required.
2. Subject-specific response: This is also known as a localized model, and localizing process is known as a calibration of the mixed-effects model [51, 57, 81, 82], which involves the measurement of a response variable (i.e., HCB in our case) for a sub-sample of trees on each sample plot and estimation of the random effects before making the HCB predictions for rest of the trees on the same sample plot. The HCB measurements from any size of sub-sample of trees can be used to estimate random effects. We used ten different alternatives, which involved the selection of differing number of trees systematically or randomly based on the total tree height on each sample plot, and estimation of the sample plot-specific random effects using the measured HCB in the validation dataset. These options are: systematically selected smallest and largest tree per sample plot (options: 1, 2), and randomly selected one to eight trees per sample plot (options: 3, 4,..,10). Since our main objective of developing the mixed-effects HCB model was for the subject-specific predictions, we evaluated the HCB predictions in the validation data using the following statistics for each sample plot [83, 84]:
  $$bias\;\%=\frac{100\;{\bar{e}}_{i}}{\bar{HCB_{i}}}\;with\;{\bar{e}}_{i}=\sum_{j=1}^{n_{i}}\frac{\left(HCB_{ij}-{\hat{HCB}}_{ij}\right)}{n_{i}}$$ (9)
  where ${\bar{e}}_{i}$ is mean prediction error for the *i*^*th*^ sample plot, *HCB*_*ij*_ and $H\hat{C}B_{ij}$ are measured and predicted height to crown base for *j*^*th*^ tree on the *i*^*th*^ sample plot, ${\bar{HCB}}_{i}$ is mean of the measured *HCB* on the *i*^*th*^ sample plot, and *n*_*i*_ is the number of observations for the *i*^*th*^ sample plot.

The random effects were estimated by applying the following empirical best linear unbiased prediction (EBLUP) method [48, 51]:

$$u_{i}=DZ_{i}^{T}{\left(Z_{i}DZ_{i}^{T}+R_{i}\right)}^{-1}\varepsilon_{i}$$ (10)

In this equation, ***u***_***i***_ is a vector of the random effect parameters (*u*_*i1*,_ *u*_*i2*_) that accounts for sample plot-level variations of HCB for *i*^*th*^ sample plot. Within-sample plot variance-covariance matrix, ***D*** was computed using Eq 6. The elements of matrix ***Z***_*i*_ are the values of partial derivatives of a nonlinear model (Eq 5) with respect to the fixed parameters associated with the random effect parameters [48, 49, 57, 81]. This matrix is thus defined by

$$\hat{Z_{i}}=\frac{\partial f\left(x_{i},\;b,u_{i}\right)}{\partial b}$$ (11)

where *b* is a vector of the fixed parameters (*b*_*1*_,.., *b*_*6*_), *u*_*j*_ is a vector of the random effect parameters (*u*_*i1*_, *u*_*i2*_), and *x*_*i*_ is a vector of the predictors on the *i*^*th*^ sample plot, and *b* is a vector of the fixed parameters.

## Results

The base model (Eq 3) only described 68% and 67% of the total variations in the HCB for Norway spruce and European beech, respectively. This this model was expanded through the integration of additional predictors: total height (H), dominant height (HDOM), basal area of trees larger in diameters than a subject tree (BAL), and basal area proportion of a species of interest (BAPOR) (Eq 5). A significant improvement in the fit statistics were seen, i.e., the expanded model with variance function included was able to describe about 79% and 75% variations on the HCB for Norway spruce and European beech, respectively. Replacing BAL with HCI further improved the model even if the model was fitted with the ordinary least squares regression (i.e., *R*^*2*^_*adj*_ ≈ 0.81 for spruce, *R*^*2*^_*adj*_ ≈ 0.78 for beech). The model fits were further improved when sample plot-level random effects were included (Table 2). There was a reduction in the unexplained variances (i.e., mean squared residuals, *σ*^*2*^) by 49% to 55% relative to that in the ordinary least square models, with more reduction in European beech. Since our main interest of developing HCB models is for the sample plot-level predictions, only results of the mixed-effects models are presented here (Table 2). For both species, spatially explicit mixed-effects model described slightly a larger part of the HCB variations than its spatially inexplicit counterpart. All parameter estimates for both spatially explicit and inexplicit mixed-effects HCB models including variance components were significantly different from zero (*p* < 0.01).

**Table 2:** Parameter estimates, variance-covariance components, and fit statistics of the mixed-effects model (Eq 5). [*R*^*2*^_*adj*_: adjusted coefficient determination; *RMSE*: root mean squared errors; *AIC*: Akaike’s information criterion; *b*_*1*_, *b*_*2*_,*…*,*b*_*6*_: fixed parameters; *u*_*j1*,_ *u*_*j2*_ = random effects parameters; *σ*^*2*^_*uj1*_: variance of *u*_*j1*_; *σ*^*2*^_*uj2*_: variance of *u*_*j2*_; *σ*^*2*^: variance according to Eq 7; *φ* = parameter of a power variance function (Eq 8); standard errors are given in the parenthesis].

Components	Parameter estimates and fit statistics			
	Norway spruce		European beech	
	Spatially explicit	Spatially inexplicit	Spatially explicit	Spatially inexplicit
Fixed				
*b*_*1*_	1.199255 (0.0421)	1.246311 (0.0422)	1.415323 (0.0711)	1.597849 (0.0682)
*b*_*2*_	0.018844 (0.0007)	0.017698 (0.0006)	0.01564 (0.00076)	0.014094 (0.00074)
*b*_*3*_	-0.05075 (0.0018)	-0.04706 (0.00175)	-0.02395 (0.00212)	-0.02886 (0.00206)
*b*_*4*_	-0.0048 (0.00187)	-0.0071 (0.00168)	-0.01955 (0.00236)	-0.01411 (0.00223)
*b*_*5*_	-0.01577 (0.00285)	-0.00486 (0.00043)	-0.014212 (0.0034)	-0.00191 (0.00058)
*b*_*6*_	-0.15772 (0.0221)	-0.10539 (0.0228)	-0.75743 (0.0388)	-0.8237 (0.0353)
Variance				
*σ*^*2*^_*uj1*_	0.8838	0.7893	0.4818	0.4457
*σ*_*uj1uj2*_	-0.01397	-0.01385	-0.00308	-0.0032
*σ*^*2*^_*uj2*_	0.000531	0.000593	0.000089	0.000129
*σ*^*2*^	0.02231	0.02318	0.02634	0.02459
Fit statistics				
*R*^*2*^_*adj*_	0.8648	0.8493	0.8509	0.8352
*RMSE*	1.9108	2.0013	2.2216	2.2704
*AIC*	10517	10663	7685	7813

**Fig 3:**
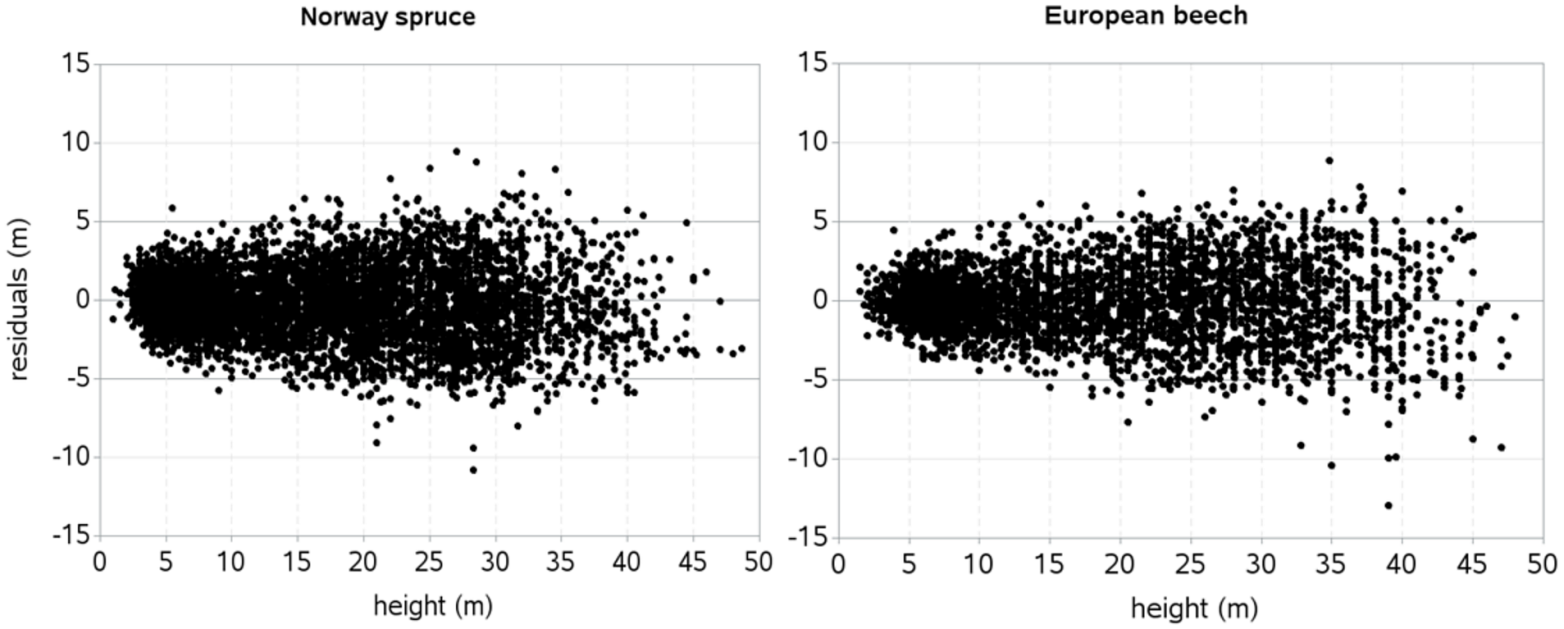
Standardized residuals of spatially inexplicit mixed-effects HCB model with a power variance function (Eq 8) included.

The heteroskedasticity in the residuals was substantially reduced through the integration of a power variance function with H as an independent variable [Eq 8, with *φ* = 0.895 (spruce), *φ* = 0.883 (beech), estimated from the data] included into the mixed-effects HCB model (Fig 3). However, small heteroskedasticity was still present for each species, and similar magnitudes of variance heteroskedasticity were also found in the spatially explicit HCB models. Among various alternatives (three variance functions applied with each of the predictors in Eq 5), none of them could reduce heteroskedasticity more effectively than that shown in Fig 3. No trend was visible in the residuals plotted against each of the predictors included in the HCB models and the potential predictors not included into the models. The histograms of the residuals showed the Gaussian distribution patterns, indicating that serious skewness in the residuals was not present. Also, no trend was visible in the residuals plotted separately for mixed species stands and monospecific stands, indicating that the mixed-effects HCB model properly fitted to the data from each stand type.

Compared to the model for Norway spruce, the model for European beech poorly fitted to data (Table 2). The effects of predictors in the model for each species also appeared significantly different. For example, the magnitudes of estimated parameters suggested that the HCB model for Norway spruce was less affected by species mixture or inter-species interactions (BAPOR) and site quality (HDOM) on HCB than those for European beech. However, there were more influences of the stand density or competitive interactions among the individual trees (BAL or HCI) on the HCB for Norway spruce than for European beech. The HCI for Norway spruce and BAPOR for European beech provided the largest contributions to the HCB model. The least contribution was provided by BAL for both species. For a given value of the total height of a tree, HCB increased with increasing site quality (increasing HDOM) and competitive interactions among the trees (increasing BAL or HCI), and decreasing species mixture or inter-species interactions (increasing BAPOR). The magnitudes of parameter estimates also suggested that the effect of HCI on HCB was significantly larger than that of BAL for each species.

**Fig 4:**
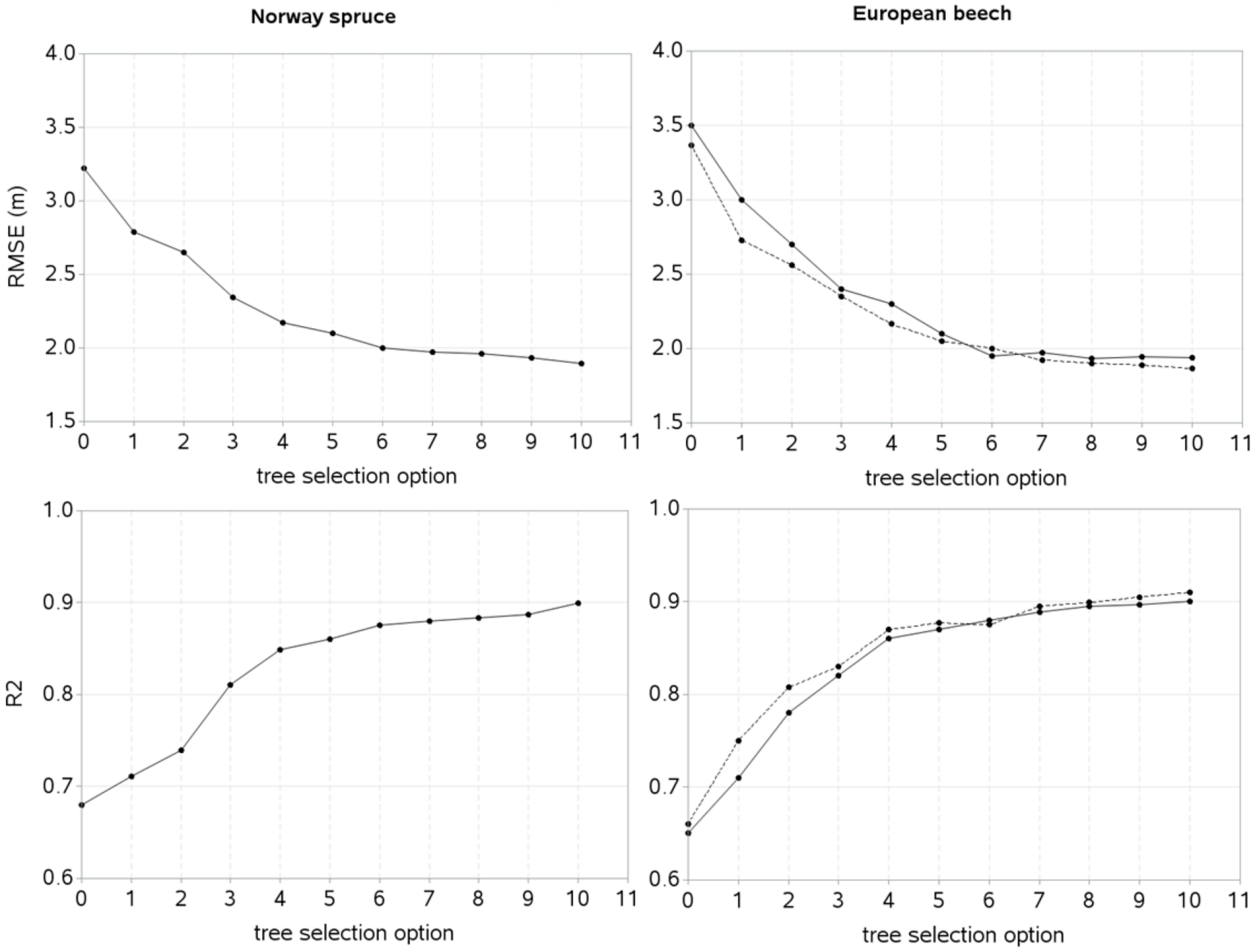
Prediction statistics of the mixed-effects HCB model for different options (1 to 10) of selecting sample trees to estimate random effects by using validation data. [Tree selection option: 0: mean response (no tree selected); 1: smallest tree; 2: largest tree; and options 3 to 10 belong to randomly selected 1 to 8 trees per sample plot, respectively; solid line: spatially inexplicit HCB model; dotted line: spatially explicit HCB model].

We tested the mixed-effects HCB models against the validation data (Fig 2). However, spatially explicit HCB model for Norway spruce could not be tested, because of missing height measurements of many trees per sample plot in the validation dataset. This did not allow us to compute the search radii (Eq 2), and consequently the competition index, HCI (Eq 1). However, both spatially explicit and spatially inexplicit HCB models for European beech were tested against the independent datasets. We applied ten alternative methods of selecting sub-sample of trees to estimate random effects and calibrate or localize the mixed-effects model. The results of the calibrated response pattern showed that the prediction accuracy increased with increasing number of sub-sample of trees used to estimate random effects (Fig 4), i.e., there was smaller RMSE and larger R^2^ relative to those of the mean response (tree selection option 0). Irrespective of the model types, the prediction accuracy steadily increased with increasing number of sample trees used to estimate random effects. But increasing trend seemed to be relatively smaller after four trees (tree selection option 6 in Fig 4). The accuracy of the mixed-effects HCB model largely depended on the method employed to selection of trees, for example, the prediction accuracy of the HCB model with the random effects estimated using one systematically selected tree (smallest or largest tree) was lower than that of the model calibrated with one randomly selected tree.

**Fig 5:**
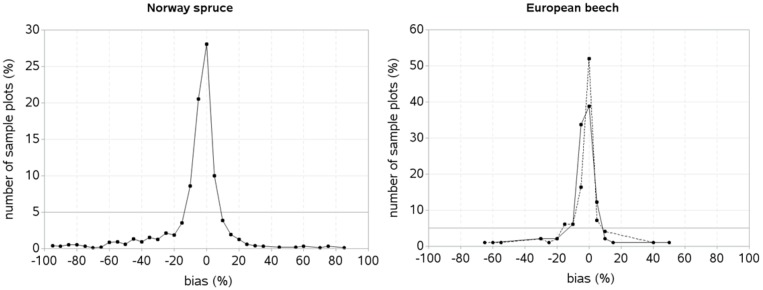
Bias of the mixed-effects HCB model in the validation data. The random effects were estimated using the measured HCB of four randomly selected trees per sample plot [solid line: spatially inexplicit HCB model; dotted line: spatially explicit HCB model].

We also analyzed the prediction bias (Eq 9) using four randomly selected trees per sample plot in the validation data. The prediction biases were found falling within ± 20% ranges for more than 95% sample plots in each species (Fig 5), indicating that the mixed-effects HCB model for most of the sample plots in the validation data worked adequately well. However, a larger bias (i.e. > 20% bias) still remained to be described for < 5% sample plots due to the influence of outlier data that originated from the heterogeneous stands. The spatially explicit HCB model for European beech showed a higher prediction accuracy than its spatially inexplicit counterpart.

## Discussion

We developed both spatially explicit and inexplicit mixed-effects HCB models using DBH and H as main predictors and species mixture, spatially explicit and inexplicit competition measures as additional predictors. Description of a large part of the HCB variations without substantial trends in the residuals (Fig 3) suggests that selection of a base model (Eq 3), predictors (Eq 5), and variance stabilizing function (Eq 8) are all suited to the data. Our HCB models are based on the extensive data that were collected from fully-mapped permanent sample plots representing a wide range of stand density, site quality, stand development stage, and management regime of the stands distributed across the Czech Republic (Fig 1). The distribution patterns of HCB and each of the six predictors in the model seem to be approximately symmetric, balanced, and regular. It is because of the large datasets collected from the stands representative to all possible stand development stages, stand densities, and site qualities, where all possible tree sizes were measured. The distribution patterns of the data could be examined or simulated using the Weibull and Gamma distribution functions. In recent years, there can be possible of using the Gamma Shape Mixture (GSM) model that correctly approximates the highly skewed distributions and precisely separates the dendrometric data from older and younger stands [85, 86].

As in other HCB modelling studies [1, 4, 11, 25, 33], where H and DBH were used as main predictors for their HCB models, we also used these variables, assuming that H and DBH better describe stand structure, tree vigor, and competition ability of the individual trees. Among various potential predictors evaluated, dominant height (HDOM), basal area of trees larger in diameters than the subject tree (BAL), basal area proportion of a species of interest (BAPOR), and Hegyi’s competition index (HCI, Eq 1) were found to have contributed more significantly to description of the HCB variations, and therefore they were selected to model individual tree HCB. Even though the prediction accuracy of the HCB models could be significantly improved by incorporating additional tree- and stand-level variables, introducing many predictors into them not only impedes the computational convergence, but also results in biased parameter estimates due to over-parameterization [50, 74]. Furthermore, including many predictors into HCB model increases the forest inventory cost. Therefore, a parsimonious model with reasonable accuracy is a preferred choice for efficient forest management [87, 88].

Application of the mixed-effects modelling approach in this study is largely justifiable, because it reduced the unexplained variances by 49%–55% relative to that in the ordinary least squares models. Furthermore, a large estimated value of the variance σ^2^_*ui1*_ for each species suggests that parameter *b*_*1*_ of the model highly varies across the sample plots. The mixed-effects models are important tools for analyzing the hierarchally structured data and their prediction accuracy would be higher than that of the ordinary least squares models [40, 48, 49, 57]. A description of slightly a larger part of the HCB variations by spatially explicit model indicates that the competition measures computed using tree positions better describes competitive interactions among the trees [37, 39, 41, 46]. A forest stand is an aggregate of the individual trees interacting over the restricted distance and such interaction largely influences tree growth, mortality, and regeneration as well [37, 89]. Quantification of the competitive interactions among the trees and inclusion of the competitive response into the HCB models can therefore be an important basis for making the informed decision.

Like other individual tree models such as crown width and crown ratio models, height-diameter models, height increment models, and HCB models [4, 23, 30, 46, 57, 65, 90], we also included HDOM as a surrogate of site index into our HCB models. The complex climatic and topographic conditions of our study area (Fig 1) might have resulted in a large variation of site quality, and consequently the relationship between HCB and HDOM largely differ among the sample plots. Growth modelling studies often show a strong relationship between HDOM and tree growth and stand development [91, 92], and its inclusion into the HCB models is therefore justifiable, because HDOM reflects site quality in terms of stand growth and yield capacity. The negative sign of the parameter estimates of HDOM is most likely due to fact that the competitive stress on the spruce stands may be much higher, so that lower large branches of the trees might have been dead by self-pruning [65].

The competitive stress, which is caused by crowding of trees, is one of the important factors affecting HCB [4, 25, 93–95]. A spatially inexplicit tree-centered measure such as BAL, which is a well-behaved density measure under all stand densities, was used to describe competition among the trees, in this study. Our result is also consistent with that from other study [27], which has shown an increased HCB with increasing competition described by BAL. The HCI also shows a similar pattern of the effect as BAL on HCB in our study (Table 2). The competitive stress and physical interactions among the branches of neighboring trees may cause the crown recession, which results in a larger HCB. The crown recession and height growth are two major phenomena affecting HCB of the trees. In a denser stand, trees of the same diameter or height usually have a larger HCB than that in the sparse stands. Various situations, such as light availability to the base of crown [96], stand density and height growth [29, 97], and physical interactions among the branches of neighboring trees [95, 98], and species composition largely influence the rate of crown recession and HCB dynamics. The increased crowding of trees results in a taller height, narrower crown, and thinner stem, but a larger HCB as a result of the crown recession [46, 95, 99].

Like other studies [39–41, 46, 100, 101], we also found that spatially explicit competition measure, i.e. HCI better described competitive interactions among the trees than spatially inexplicit measure, and this is the reason why a spatially explicit model better described both model fitting and validation data for each species (Table 2, Figs 4 and 5). Spatially explicit competition measure describes competitive stress significantly differently as competition largely varies with stand density, species and size of individuals, site quality, and slope of a sample plot [39, 102, 103]. Inclusion of species-specific competition effects [36, 69, 89, 104], and other characteristics rather than tree dimensions into the spatially explicit index is rarely practiced, because computational complexity increases with increasing number of variables in the index. We did not include the effects of species and other characteristics in the HCI (Eq 1) in order to make our HCB models simpler and more applicable. Application of the spatially explicit models needs tree coordinates, which may not be recorded from the routine forest inventories because of a higher cost. It might become cheaper when spatial data from airborne laser scanning [105] or data generated based on the empirical spatial distribution patterns of the trees [106] would be available.

The effect of species mixture (BAPOR) on the HCB model is highly significant and similar effects on tree growth and stand characteristics have been frequently reported [42, 43, 46, 57]. The negative sign of parameter estimates of BAPOR (Table 2) suggests that, for a given site quality and stand density, more the number of tree species present in a stand, competition among the trees would be higher, the rate of crown recession would be higher, and consequently larger would be the HCB. In recent decades, the forest managers are more interested to the multi-aged and mixed species stands than to the even-aged and monospecific stands. This has encouraged forest researchers to analyze the effects of species mixture on tree growth and stand dynamics [42, 43, 45–47, 57, 107, 108]. All these studies have shown that species mixture in a stand creates the neighborhood situation where patterns of canopy space filling, resource supply, resource capture, and resource use efficiency would be more favorable for tree growth than those in a monospecific stand. The knowledge of species mixing effects is useful for the informed decision-making in forestry.

We examined ten different methods of determining the optimum size of sample trees used for estimating random effects in the validation data (Fig 4). The mixed-effects HCB model predicted HCB with a reasonable accuracy, but large errors still remained to be accounted for less than 5% sample plots (Fig 5). This is mainly due to outlier observations originated from the trees in the extreme stand densities. The prediction accuracy of the localized mixed-effects HCB model depends largely on the stand structure of a sample plot, number of trees, and representativeness of HCB of chosen trees for estimating random effects. For sample plot with homogenous stand structure, one or two trees may work adequately well [82, 84], but more trees are needed for heterogeneous stand structure to ensure a higher prediction accuracy. Our study shows that the localized mixed-effects HCB model has a higher prediction accuracy relative to that of the mean response even when one randomly selected tree or the smallest or largest tree per sample plot was used to estimate random effects. Also, the rate of reduction of the predction errors was too small to be insignificant when more than four trees were used to estimate random effects. It is likely that many variables influencing HCB may not be known or measured practically, but their effects could be captured by few trees per sample plot, which substantially improves the prediction accuracy [4, 50]. Our findings are also consistent to those of the crown width, HCB and height-diameter modelling studies [4, 49, 50, 57, 109–111], where a small reduction in the prediction errors was found after more than four trees per sample plot were used to estimate random effects. A sample of more than four trees may not be justifiable because of increased sampling cost with a little gain in the prediction accuracy. Thus, four trees per sample plot can be an optimum number to ensure a higher prediction accuracy of the localized mixed-effects models [4, 50, 110, 111].

The fitting and prediction behaviors of the HCB model for each species seems significantly different (Table 2, Figs 4 and 5). Compared to the model for Norway spruce, the model for European beech poorly fitted to data, but its predicting performance seems relatively better (Figs 4 and 5). However, because of lack of external independent data, prediction behavior of the HCB model for European beech could not be properly evaluated. The poorer fitting of the model to the data for European beech may be due to larger variation of the HCB data relative to that of Norway spruce (Fig 2). The effects of the predictors for each tree species also appear significantly different, for example, HCB for Norway spruce is less affected by species mixture or inter-species interactions and site quality than for European beech. The effect of competition on HCB seems higher for Norway spruce and competitive stress described by spatially explicit competition measure (HCI) is significantly higher for this species, because of its more complex and heterogeneous stand structures, where the competition measures computed using the spatial position of trees describe competitive interactions among the trees most effectively [36, 37, 39–41, 46].

## Conclusion

A spatially explicit mixed-effects model showed more attractive fit statistics than those of the spatially inexplicit counterpart even though their difference was small in each species. The model users may therefore prefer spatially inexplicit mixed-effects HCB model for application, as this model does not require the Hegyi’s competition index as an input, which is computationally more complex than basal area of trees larger in diameters than a subject tree. However, when a detailed description of the stands and higher prediction accuracy is required, application of the spatially explicit mixed-effects HCB model could obviously be a preferred choice. A test of the mixed-effects HCB model with the random effects estimated using four trees per sample plot in the validation data confirmed that the model was accurate enough for the prediction of HCB for a wide range of tree size, site quality, stand density, and species mixture. Measuring HCB of at least four randomly selected trees of a species of interest per sample plot is therefore recommended for localizing the mixed-effects HCB model and predicting HCB of remaining trees on the plot. Growth simulations can be made from the data that lack the values for either crown ratio or HCB using HCB models.

## Supporting information

S1 Table Training and validation datasets. (ZIP)
